# Predictors of pathological complete response to neoadjuvant treatment and changes to post-neoadjuvant HER2 status in HER2-positive invasive breast cancer

**DOI:** 10.1038/s41379-021-00738-5

**Published:** 2021-02-01

**Authors:** Ayaka Katayama, Islam M. Miligy, Sho Shiino, Michael S. Toss, Karim Eldib, Sasagu Kurozumi, Cecily M. Quinn, Nahla Badr, Ciara Murray, Elena Provenzano, Grace Callagy, Cian Martyn, Rebecca Millican-Slater, Colin Purdie, Dave Purnell, Sarah E. Pinder, Tetsunari Oyama, Abeer M. Shaaban, Ian Ellis, Andrew H. S. Lee, Emad A. Rakha

**Affiliations:** 1 Nottingham Breast Cancer Research Centre, Division of Cancer and Stem Cells, School of Medicine, Nottingham City Hospital, University of Nottingham, Nottingham, UK; 2 Diagnostic Pathology, Gunma University Graduate School of Medicine, Maebashi, Japan; 3 Department of Pathology, Faculty of Medicine, Menoufia University, Shebin El-Kom, Egypt; 4 Department of Breast Surgery, National Cancer Centre Hospital, Tokyo, Japan; 5 Department of Histopathology, Nottingham University Hospitals, Nottingham, UK; 6 Department of Breast Surgery, International University of Health and Welfare, Narita, Japan; 7 Department of General Surgical Science, Gunma University Graduate School of Medicine, Maebashi, Japan; 8 Department of Histopathology, St. Vincent’s University Hospital, Dublin, and School of Medicine, University College Dublin, Dublin, Ireland; 9 Institute of Cancer and Genomic Sciences, The University of Birmingham, Edgebaston, Birmingham, UK; 10 Department of Histopathology, Cambridge University NHS Foundation Trust, Cambridge, UK; 11 Discipline of Pathology, School of Medicine, Lambe Institute for Translational Research, NUI Galway, Galway, Ireland; 12 Department of Histopathology, St James’s University Hospital, Leeds, UK; 13 Department of Breast Pathology, Ninewells Hospital and Medical School, Dundee, UK; 14 Histopathology department, University Hospitals of Leicester, Leicester, UK; 15 Division of Cancer Studies, King’s College London, Guy’s Hospital, London, UK

**Keywords:** Targeted therapies, Breast cancer, Chemotherapy

## Abstract

The response of human epidermal growth factor receptor2 (HER2)- positive breast cancer (BC) patients to anti-HER2 targeted therapy is significant. However, the response is not uniform and a proportion of HER2-positive patients do not respond. This study aims to identify predictors of response in the neoadjuvant treatment and to assess the discordance rate of *HER2* status between pre- and post-treatment specimens in HER2-positive BC patients. The study group comprised 500 BC patients treated with neoadjuvant chemotherapy (NACT) and/or neoadjuvant anti-HER2 therapy and surgery who had tumours that were 3+ or 2+ with HER2 immunohistochemistry (IHC). HER2 IHC 2+ tumours were classified into five groups by fluorescence in situ hybridisation (FISH) according to the 2018 ASCO/CAP guidelines of which Groups 1, 2 and 3 were considered *HER2* amplified. Pathological complete response (pCR) was more frequent in HER2 IHC 3+ tumours than in HER2 IHC 2+/*HER2* amplified tumours, when either in receipt of NACT alone (38% versus 13%; *p* = 0.22) or neoadjuvant anti-HER2 therapy (52% versus 20%; *p* < 0.001). Multivariate logistic regression analysis showed that HER2 IHC 3+ and histological grade 3 were independent predictors of pCR following neoadjuvant anti-HER2 therapy. In the HER2 IHC 2+/*HER2* amplified tumours or ASCO/CAP FISH Group 1 alone, ER-negativity was an independent predictor of pCR following NACT and/or neoadjuvant anti-HER2 therapy. In the current study, 22% of HER2-positive tumours became HER2-negative by IHC and FISH following neoadjuvant treatment, the majority (74%) HER2 IHC 2+/*HER2* amplified tumours. Repeat HER2 testing after neoadjuvant treatment should therefore be considered.

## Introduction

Approximately 15% of invasive breast cancers (BCs) are *human epidermal growth factor receptor2 (HER2)* positive, defined as showing *HER2* gene amplification or protein overexpression, and such tumours have been shown to be sensitive to anti-HER2 targeted therapy [1–3]. Currently, a combination of sequential chemotherapy and anti-HER2 therapy is the standard treatment for HER2-positive BC both in the neoadjuvant and adjuvant setting [4]. Immunohistochemistry (IHC) and/or in situ hybridisation (ISH) is routinely used to evaluate the HER2 status for treatment selection.

The current American Society of Clinical Oncology/College of American Pathologists (ASCO/CAP) guidelines regard HER2 IHC score 3+ as positive, score 2+ as equivocal and scores 0 and 1+ as negative [5]. If the IHC result is score 2+, such patients are tested for *HER2* amplification by ISH; most commonly fluorescence in situ hybridisation (FISH), to assess the average *HER2* gene and chromosome enumeration probe 17 (CEP17) copy numbers (CNs) per carcinoma cell and the ratio of these [5]. The 2018 ASCO/CAP guidelines divide *HER2* FISH status into five groups (Table 1) [5].

**Table 1:** HER2 FISH groups in 2018 ASCO/CAP guidelines.

Group	HER2/CEP17 ratio	Average *HER2* gene CN
1	≥2.0	≥4.0
2	≥2.0	<4.0
3	<2.0	≥6.0
4	<2.0	≥4.0 and <6.0
5	<2.0	<4.0

*CN* copy number.

For HER2-positive BC, defined as either IHC 3+ or IHC 2+ with *HER2* amplification, neoadjuvant chemotherapy (NACT) plus neoadjuvant anti-HER2 therapy is an effective treatment option [4]. The pathological complete response (pCR) rate at the time of surgery is commonly used as an endpoint in clinical trials and a predictor of good prognosis in HER2-positive BC with neoadjuvant treatment [6–8]. The most widely-agreed definition of pCR is no residual invasive carcinoma both in the breast and axillary lymph nodes regardless of the presence of residual ductal carcinoma in situ (DCIS) (ypT0/is ypN0) [8].

Retrospective studies of HER2-positive BC patients who have received NACT plus neoadjuvant anti-HER2 therapy have reported a higher rate of pCR in IHC 3+ compared to IHC 2+/*HER2* amplified tumours [9]. However, little attention has been paid to predictors of pCR among the different ASCO/CAP FISH groups. In addition, some clinical trials showed that pCR rates are lower in oestrogen receptor (ER)-positive/HER2-positive BC than in ER-negative/HER2-positive BC [10–12]. It remains unclear if this holds true for the different ASCO/CAP FISH amplified groups.

Meanwhile, others studies have reported discordant HER2 status between the pre-treatment biopsy and the post-treatment surgical specimen [13–18]. Loss of HER2-positivity in the residual tumour has been reported to be associated with a poorer outcome compared to tumours that remain HER2-positive following neoadjuvant treatment [16–18]. The change in HER2 status may also affect the selection of post-neoadjuvant treatment. However, there is no consensus on whether or not repeat HER2 testing in post-neoadjuvant residual disease should be performed in routine practice.

In this study, we have analysed 500 invasive BCs with HER2 IHC 3+, or IHC 2+ for which HER2 FISH data were available, from patients who received NACT and/or neoadjuvant anti-HER2 therapy with subsequent therapeutic surgery. Our aim was to evaluate the relationship between HER2 categories and pCR alongside other variables. We also report the level of concordance for *HER2* and the ASCO/CAP FISH groups between the pre- and post-treatment specimens in this study cohort.

## Materials and methods

### Study cohort

A total of 500 invasive BCs with HER2 IHC 3+ or IHC 2+ for which HER2 FISH data were available from patients who received NACT and/or neoadjuvant anti-HER2 therapy with subsequent therapeutic surgery between 2013 and 2020 were included. Exclusion criteria were: (1) no information of *HER2* gene CN or *HER2*/CEP17 ratio in pre-treatment specimens for IHC 2+ tumours; (2) patients treated with neoadjuvant hormonal therapy alone; (3) lack of data on pathological response in the surgical specimen. Therefore 75 of the 575 patients were excluded. The majority of patients were treated at Nottingham University Hospitals NHS Trust, Nottingham (*n* = 254) with additional patients from the following nine institutions: Addenbrookes Hospital, Cambridge; University Hospitals Birmingham NHS Foundation Trust; University Hospitals of Leicester NHS Trust; St. Vincent’s University Hospital, Dublin; University Hospital Galway, Galway; Burney Breast Unit, St Helens and Knowsley Teaching Hospital NHS Trust, Liverpool; Guy’s and St Thomas’ NHS Foundation Trust, London; Ninewells Hospital, Dundee; University of Turin, Turin Italy. Patients with tumours that demonstrated an HER2 IHC score of 2+ but that were non-amplified with FISH were included as a control group (*n* = 151).

Patients were considered eligible for anti-HER2 therapies if their tumours showed a HER2 IHC score of 3+, or 2+ with a ratio ≥2.0 regardless of the *HER2* CN or if the *HER2* gene CN was ≥6, as recommended by UK guidelines (corresponding to ASCO/CAP FISH Groups 1, 2 and 3) [19]. Treatment was given according to institutional guidelines. Exact neoadjuvant regimens and number of cycles varied slightly but patients were divided into four groups according to the neoadjuvant treatment received: chemotherapy alone; chemotherapy with trastuzumab; chemotherapy with dual anti-HER2 agents (i.e. trastuzumab with either pertuzumab or lapatinib) and anti-HER2 therapy alone.

pCR was defined as no residual invasive carcinoma in both breast and axillary lymph nodes regardless of the presence of residual DCIS (ypT0/is ypN0) [8]. Histological grade was evaluated according to the Nottingham modification of the Scarff-Bloom-Richardson system on the pre-treatment specimens [20]. All of the histopathological data used in the analysis were derived from the original pathology reports.

### Immunohistochemistry and FISH assay

IHC for ER and progesterone receptor (PR), and both HER2 IHC and FISH for HER2 in pre- and post-treatment specimens were assessed as per UK guidelines [19, 21]. HER2 IHC was scored as positive (3+), equivocal (2+) or negative (1+/0), and IHC score 2+ patients were tested for *HER2* amplification by FISH [19]. As per the 2018 ASCO/CAP guidelines, *HER2* FISH status was assigned to one of five groups (Table 1) [5]. However, it was impossible to completely follow the recommendation for concomitant IHC review and reassessment and recounting of FISH slides in the less common FISH patterns (Groups 2, 3 and 4) owing to the retrospective nature of this study. In this study, ASCO/CAP FISH Groups 1, 2 and 3 were defined as *HER2* amplified according to UK guidelines, which differ from 2018 ASCO/CAP guidelines where Group 2 tumours are now considered non-amplified [19]. According to CEP17 CN, chromosome 17 (chr 17) status was defined as monosomy of chr17 (m17) if <1.5, normal chr17 (n17) if ≥1.5 but <3.0, and polysomy of chr17 (p17) if ≥3.0 average CN per carcinoma cells [22, 23]. For ER and PR, tumours were classified as positive when there was ≥1 % nuclear staining in invasive carcinoma cells [21].

### Statistical analysis

Statistical analysis was performed using EZR (Saitama Medical Center Jichi Medical University; http://www.jichi.ac.jp/saitama-sct/SaitamaHP.files/statmed.html), which is a graphical user interface for R (The R Foundation for Statistical Computing, Vienna, Austria, version 2.13.0) [24]. Associations between clinicopathological variables and pCR were examined with Fisher’s exact tests or Pearson’s χ^2^ test, as appropriate. A logistic regression model was applied to evaluate the effect of covariates on pCR. If a variable remained at a level of *p* value ≤ 0.15, it was incorporated into the final multivariable model [25]. Comparison between pre- and post-treatment receptor status was assessed by McNemar’s test. A *p* value ≤ 0.05 was considered statistically significant. This study was approved by the Nottingham Research Tissue Bank Access Committee under the IRAS Project ID: 184265. Data collected were fully anonymised.

## Results

### Patient characteristics

Table 2 shows the demographic and treatment characteristics of the study cohort by HER2 categories. The median age at diagnosis was similar amongst all the groups. The tumour histological type was most commonly invasive breast carcinoma of no special type (NST), whilst histological grade was predominantly 2 or 3 across the whole cohort. ER and PR were both more often negative in IHC 3+ tumours than in other groups (*p* < 0.001). As expected, patients with HER2-positive tumours were significantly more likely to receive anti-HER2 therapy (*p* < 0.001). Chemotherapy with trastuzumab was the main neoadjuvant treatment in patients with tumours that were HER2 IHC 3+ or IHC 2+ within the *HER2* amplified group (ASCO/CAP FISH Groups 1, 2 and 3), while all patients within the *HER2* non-amplified group (Groups 4 and 5) received chemotherapy alone. An anthracycline- and taxane-based chemotherapy regimen was received by the majority of patients across all groups, but the number of patients treated without an anthracycline regimen was significantly higher in Group 1 (*p* < 0.001).

**Table 2:** Patients baseline characteristics.

Characteristic	IHC3+ (*n* = 180) No. (%)	IHC 2+ (*n* = 320)					*P* value
		Group 1 (*n* = 112) No. (%)	Group 2 (*n* = 48) No. (%)	Group 3 (*n* = 9) No. (%)	Group 4 (*n* = 55) No. (%)	Group 5 (*n* = 96) No. (%)	
Age							
Median [range]	52 [23–83]	52 [27–86]	52 [27–78]	50 [36–71]	54 [23–73]	53 [26–75]	
Histology type							
Ductal, NST	160 (88.9)	97 (86.6)	43 (89.6)	9 (100)	48 (87.3)	83 (86.5)	0.45
Special types	7 (3.9)	8 (7.1)	2 (4.2)	0	4 (7.3)	1 (1.0)	
Lobular	6 (3.3)	6 (5.4)	2 (4.2)	0	3 (5.4)	9 (9.4)	
Mixed (ductal and lobular)	4 (2.2)	1 (0.9)	0	0	0	3 (3.1)	
Unknown	3 (1.7)	0	1 (2.0)	0	0	0	
Histological grade							
1	2 (1.1)	1 (0.9)	1 (2.1)	0	1 (1.8)	2 (2.1)	0.17
2	103 (57.2)	55 (49.1)	26 (54.2)	3 (33.3)	25 (45.5)	64 (66.7)	
3	64 (35.6)	52 (46.4)	20 (41.6)	6 (66.7)	29 (52.7)	28 (29.1)	
Unknown	11 (6.1)	4 (3.6)	1 (2.1)	0	0	2 (2.1)	
ER							
Positive	103 (57.2)	87 (77.7)	35 (72.9)	9 (100)	44 (80.0)	72 (75.0)	**<0.001**
Negative	75 (41.7)	25 (22.3)	13 (27.1)	0	11 (20.0)	24 (25.0)	
Unknown	2 (1.1)	0	0				
PR							
Positive	69 (38.3)	51 (45.5)	21 (43.8)	7 (77.8)	28 (50.9)	53 (55.2)	**<0.001**
Negative	110 (61.1)	37 (33.1)	15 (31.2)	2 (22.2)	15 (27.3)	36 (37.5)	
Unknown	1 (0.6)	24 (21.4)	12 (25.0)	0	12 (21.8)	7 (7.3)	
Neoadjuvant treatments							
Chemotherapy alone	16 (8.9)	8 (7.1)	6 (12.5)	2 (22.2)	55 (100)	96 (100)	**<0.001**
Chemotherapy+anti-HER2 therapy (single)	134 (74.4)	87 (77.7)	37 (77.1)	5 (55.6)	0	0	
Chemotherapy+dual anti-HER2 therapy	27 (15.0)	13 (11.6)	3 (6.2)	1 (11.1)	0	0	
Anti-HER2 therapy alone	3 (1.7)	4 (3.6)	2 (4.2)	1 (11.1)	0	0	
Chemotherapy regimens							
Anthracyclines and Taxanes	128 (72.3)	66 (61.1)	36 (78.3)	5 (62.5)	44 (80.0)	75 (78.1)	**<0.001**
Anthracyclines without Taxanes	32 (18.1)	6 (5.6)	2 (4.3)	1 (12.5)	6 (10.9)	17 (17.7)	
Non-anthracyclines	17 (9.6)	36 (33.3)	8 (17.4)	2 (25.0)	5 (9.1)	4 (4.2)	

According to 2018 ASCO/CAP guidelines, HER2 FISH status were divided into five groups in IHC 2+ patients: group 1, HER2/CEP17 ratio ≥ 2.0, average HER2 gene CN ≥ 4.0; group 2, HER2/CEP17 ratio ≥ 2.0, HER2 gene CN < 4.0; group 3, HER2/CEP17 ratio < 2.0, HER2 gene CN > 6.0; group 4, HER2/CEP17 ratio < 2.0, average HER2 gene CN ≥ 4.0 and ≤6.0; and group 5, HER2/CEP17 ratio < 2.0, HER2 gene CN < 4.0.

Bold values indicate statistical significance *p* < 0.05.

*IHC* immunohistochemistry, *NST* no special type.

### HER2 categories and pathologic complete response

**Fig. 1:**
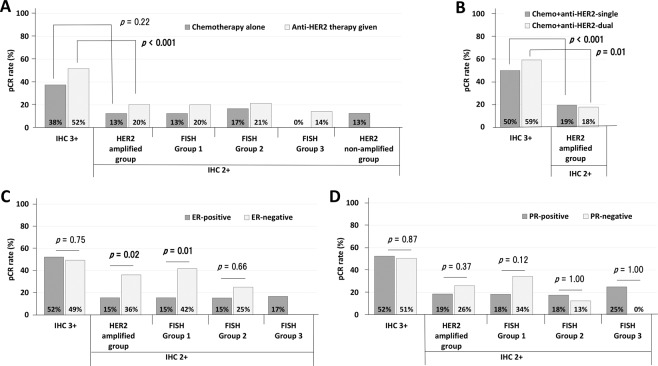
The pathological complete response rate in HER2 categories. (**A**) according to types of neoadjuvant treatment for whole patients, (**B**) different anti-HER2 therapies for HER2-positive patients, (**C**) ER status for anti-HER2 therapy given patients, and (**D**) PR status for anti-HER2 therapy given patients.

Comparisons of pCR rate within the HER2 categories were made according to ER and PR status and whether anti-HER2 therapy was given (Fig. 1). For whole patients, relationship between treatment regimens and pCR rate among various HER2 categories was different (Fig. 1A). For IHC 3+ tumours, the pCR rate was 37.5% (*n* = 6/16) for patients treated with chemotherapy alone and 51.8% (85/164) following anti-HER2 therapy. Among IHC 2+ tumours, the pCR rate following chemotherapy alone was as follows: 12.5% (2/16) in the *HER2* amplified group (Groups 1, 2 and 3); 12.5% (1/8) in Group 1; 16.7% (1/6) in Group 2; 0% (0/2) in Group 3; 12.6% (19/151) in the *HER2* non-amplified group (Groups 4 and 5). The pCR rate in IHC 2+ tumours when anti-HER2 therapy was also given was as follows: 20.3% (31/153) in *HER2* amplified tumours (Groups 1, 2 and 3); 20.2% (21/104) in Group 1; 21.4% (9/42) in Group 2; and 14.3% (1/7) in Group 3. Thus, IHC 3+ tumours had higher rates of pCR than IHC 2+/*HER2* amplified tumours, when either in receipt of chemotherapy alone (37.5% versus 12.5%; *p* = 0.22) or anti-HER2 therapy (51.8% versus 20.3%; *p* < 0.001).

We then evaluated the likelihood of pCR following different anti-HER2 therapies (chemotherapy plus single anti-HER2 therapy or chemotherapy plus dual anti-HER2 therapy) for HER2-positive tumours (Fig. 1B). Whether treated with chemotherapy plus single or chemotherapy plus dual anti-HER2 therapy, patients with IHC 3+ tumours had a significantly higher pCR rate than those with the IHC 2+/*HER2* amplified tumours (pCR rate in chemo+anti-HER2-single, 50.0% in IHC 3+ versus 19.4% in IHC 2+/*HER2* amplified; *p* < 0.001) (pCR rate in chemo+anti-HER2-dual, 59.3% in IHC 3+ versus 17.6% in IHC 2+/*HER2* amplified; *p* = 0.01). For IHC3+ tumours, chemotherapy plus dual anti-HER2 therapy showed higher pCR rate than chemotherapy plus single anti-HER2 therapy (59.3% versus 50.0%; *p* = 0.41).

For patients who received anti-HER2 therapy (Fig. 1C, D), those who had ER-negative tumours showed a significantly higher pCR rate than those who had ER-positive tumours in Group 1 (*p* = 0.01). PR status did not significantly affect the pCR rate.

### Prediction of pCR according to HER2 categories

The association between clinicopathological and treatment parameters and the attainment of a pCR was examined in the whole cohort, in the different HER2 categories and in those who received anti-HER2 therapy by univariate and multivariate stepwise regression model (Table 3). Within the cohort of patients with IHC 3+ tumours, pCR was not associated with any additional factor studied. Among those with IHC 2+/*HER2* amplified tumours (Groups 1, 2 and 3), ER-negativity was identified as an independent predictor of pCR (ER negative versus positive; OR, 11.80; 95%CI, 1.38–101.00; *p* = 0.02) which remained the case in Group 1 alone (ER negative versus positive; OR, 3.71; 95% CI, 1.28–10.70; *p* = 0.02). In Group 2, histological grade 3 was an independent predictor of pCR (3 versus 1, 2; OR, 7.86; 95% CI, 1.39–44.40; *p* = 0.02). For patients treated with anti-HER2 therapy, histological grade 3 (3 versus 1, 2; OR, 1.750; 95% CI, 1.02–3.01; *p* = 0.04) and HER2 IHC 3+(IHC 3+ versus IHC 2+/*HER2* amplified; OR, 3.490; 95% CI, 1.98–6.16; *p* < 0.001) were identified as independent predictors of pCR.

**Table 3:** Univariate and multivariate logistic regression model for pCR according to HER2 categories or anti-HER2 therapy given.

Parameters	Risk/reference	Univariant analysis					Multivariant analysis		
		pCR/non-pCR No. (%)	pCR/non-pCR No. (%)	OR	95% CI	*P* value	OR	95% CI	*P* value
IHC 3+									
Age	≥50/<50	47/54 (46.5)	42/34 (49.4)	0.71	0.39–1.28	0.29	–	–	–
Histological grade	3/1, 2	36/28 (56.2)	47/58 (62.7)	1.59	0.85–2.97	0.16	–	–	–
ER	Negative/positive	38/37 (50.6)	51/52 (49.5)	1.05	0.58–1.90	1	–	–	–
PR	Negative/positive	57/53 (51.8)	33/36 (47.8)	1.17	0.64–2.14	0.65	–	–	–
Anti-HER2 therapy	Yes/No	85/79 (51.8)	6/10 (37.5)	1.79	0.62–5.16	0.31	–	–	–
Chemotherapy regimens	Anthracyclines/Non-anthracyclines	81/79 (50.6)	8/9 (47.1)	1.15	0.42–3.14	0.80	–	–	–
*HER2* amplified group (Groups 1, 2 and 3)									
Age	≥50/<50	19/81 (19.0)	14/54 (20.6)	0.91	0.42–1.96	0.85	–	–	–
Histological grade	3/1, 2	23/55 (29.5)	10/76 (11.6)	3.18	1.40–7.21	**0.006**	2.10	0.83–5.36	0.12
ER	Negative/positive	15/23 (39.5)	18/113 (13.7)	4.09	1.81–9.29	**<0.001**	11.80	1.38–101.00	**0.02**
PR	Negative/positive	15/39 (27.8)	13/66 (16.5)	1.95	0.84–4.53	0.133	0.252	0.03–2.07	0.20
Anti-HER2 therapy	Yes/No	31/122 (20.3)	2/14 (12.5)	1.78	0.38–8.24	0.74	–	–	–
Chemotherapy regimens	Anthracyclines/Non-anthracyclines	20/96 (17.2)	10/36 (21.7)	0.75	0.32–1.76	0.51	–	–	–
FISH Group 1									
Age	≥50/<50	14/52 (21.2)	8/38 (17.4)	1.28	0.49–3.35	0.81	–	–	–
Histological grade	3/1, 2	14/38 (26.9)	8/48 (14.3)	2.21	0.84–5.82	0.15	1.58	0.56–4.45	0.39
ER	Negative/positive	10/15 (40.0)	12/75 (13.8)	4.17	1.52–11.40	**0.008**	3.71	1.28–10.70	**0.02**
PR	Negative/positive	11/26 (29.7)	9/42 (17.6)	1.97	0.72–5.41	0.21	–	–	–
Anti-HER2 therapy	Yes/No	21/83 (20.2)	1/7 (12.5)	1.77	0.21–15.20	0.60	–	–	–
Chemotherapy regimens	Anthracyclines/Non-anthracyclines	12/60 (16.7)	9/27 (25.0)	0.6	0.23–1.59	0.31	–	–	–
FISH Group 2									
Age	≥50/<50	4/25 (13.8)	6/13 (31.6)	0.35	0.08–1.45	0.16	–	–	–
Histological grade	3/1, 2	8/12 (40.0)	2/25 (7.4)	8.33	1.53–45.40	**0.01**	7.86	1.39–44.40	**0.02**
ER	Negative/positive	5/8 (38.5)	5/30 (14.3)	3.75	0.87–16.20	0.11	3.27	0.66–16.30	0.15
PR	Negative/positive	4/11 (26.7)	3/18 (14.3)	2.18	0.41–11.60	0.42	–	–	–
Anti-HER2 therapy	Yes/No	9/33 (21.4)	1/5 (16.7)	1.36	0.14–13.20	1.00	–	–	–
Chemotherapy regimens	Anthracyclines/Non-anthracyclines	7/31 (18.4)	1/7 (12.5)	1.58	0.17–15.00	1.00	–	–	–
Anti-HER2 therapy given									
Age	≥50/<50	59/121 (32.8)	55/78 (41.4)	0.69	0.43–1.10	0.12	0.71	0.42–1.22	0.22
Histological grade	3/1, 2	55/78 (41.4)	53/116 (31.4)	1.54	0.96–2.48	0.09	1.75	1.02–3.01	**0.04**
ER	Negative/positive	47/57 (45.2)	67/144 (31.8)	1.77	1.09–2.87	**0.02**	1.34	0.64–2.82	0.43
PR	Negative/positive	64/85 (43.0)	46/88 (34.3)	1.44	0.89–2.33	0.15	1.07	0.52–2.21	0.86
HER2	3 +/2+	85/79 (51.8)	31/122 (20.3)	4.23	2.57–6.98	**<0.001**	3.49	1.98–6.16	**<0.001**
Chemotherapy regimens	Anthracyclines/Non-anthracyclines	93/152 (38.0)	18/44 (29.0)	1.50	0.82–2.74	0.24	–	–	–

According to 2018 ASCO/CAP guidelines, *HER2* FISH status were divided into five groups in IHC 2+ patients: group 1, *HER2*/CEP17 ratio ≥ 2.0, average *HER2* gene CN ≥ 4.0; group 2, *HER2*/CEP17 ratio ≥ 2.0, *HER2* gene CN < 4.0; group 3, *HER2*/CEP17 ratio < 2.0, *HER2* gene CN > 6.0; group 4, *HER2*/CEP17 ratio < 2.0, average *HER2* gene CN ≥ 4.0 and <6.0; and group 5, *HER2*/CEP17 ratio < 2.0, *HER2* gene CN < 4.0.

Bold values indicate statistical significance *p* < 0.05.

*IHC* immunohistochemistry, *FISH* fluorescence in situ hybridisation, *pCR* pathological complete response, *OR* odds ratio, *CI* confidence interval.

### Changes in ER, PR and HER2 status after neoadjuvant treatment

HER2 status was assessed in the residual invasive carcinoma present after NACT and/or neoadjuvant anti-HER2 therapy in 221 patients. For comparison any ER and PR status changes were also assessed (143, 140 patients respectively) (Table 4). Of the 139 patients with pre-treatment HER2-positive tumours (IHC 3+ or IHC 2+/*HER2* amplified), 31 tumours (22.3%) become HER2-negative (IHC 2+/*HER2* non-amplified, or IHC 0/1+) after treatment, whereas 13/82 (15.9%) of patients with HER2-negative tumours before treatment changed to HER2-positive following treatment (*p* = 0.01). In our cohort, neoadjuvant treatment regimens were not significantly associated with changes to post-neoadjuvant HER2 categories (data not shown). None of the 101 ER-positive tumours before treatment changed to ER-negative after treatment, whereas 4/42 (9.5%) of patients with pre-treatment ER-negative tumours changed to ER-positive. Of the 76 patients with PR-positive tumours before treatment, 15 (19.7%) had PR-negative tumours after treatment, whereas 11/64 (17.2%) patients with PR-negative tumours before treatment changed to PR-positive. These differences were not significant (*p* > 0.05).

**Table 4:** Comparison of HER2, ER and PR status between the pre-treatment core biopsy and the post-treatment excision specimen.

Pre-treatment	Total no. (%)	Post-treatment		*P* value
		Positive no. (%)	Negative no. (%)	
HER2				
Positive	139 (62.9)	108 (77.7)	**31 (22.3)**	**0.01**
Negative	82 (37.1)	13 (15.9)	69 (84.1)	
ER				
Positive	101 (70.6)	101 (100)	0	0.13
Negative	42 (29.4)	4 (9.5)	38 (90.5)	
PR				
Positive	76 (54.3)	61 (80.3)	15 (19.7)	0.56
Negative	64 (45.7)	11 (17.2)	53 (82.8)	

Bold values indicate statistical significance *p* < 0.05.

We then evaluated HER2 concordance between pre- and post-treatment specimens according to the different HER2 categories (Table 5). The highest level of concordance (78.4%) was observed for IHC3 + tumours followed by 55.2% in ASCO/CAP FISH Group 2. No tumours within ASCO/CAP FISH Groups 1, 2 or 3 in the pre-treatment specimen were assessed IHC 3+ in the post-treatment excision specimen. Focussing on the 5 ASCO/CAP FISH groups, 62 patients (45.6%) showed a change in the FISH group pre- and post-treatment; 9.6% of *HER2* amplified tumours (Groups 1, 2 and 3) changed FISH group whilst, 16.9% of *HER2* non-amplified tumours (Groups 4 and 5) changed. The ASCO/CAP FISH group was unchanged in 54.4% of patients. Importantly, 38.1% (16/42) of tumours that were identified as being within Group 1 in the pre-treatment specimen changed to *HER2* non-amplified group in the excision specimen.

**Table 5:** Correlation between HER2 categories in pre-treatment core biopsy and the post-treatment excision specimen.

Pre-treatment	Total no. (%)	Post-treatment						
		IHC 3+ No. (%)	IHC 2+					IHC 1+ or 0
			Group 1 no. (%)	Group 2 no. (%)	Group 3 no. (%)	Group 4 no. (%)	Group 5 no. (%)	
IHC 3+	65 (29.4)	**51 (78.4)**	4 (6.2)	2 (3.0)	0	0	4 (6.2)	4 (6.2)
IHC 2+								
Group 1	42 (19.0)	0	**21** (**50.0)**	5 (11.9)	0	4 (9.5)	12 (28.6)	0
Group 2	29 (13.1)	0	7 (24.1)	**16 (55.2)**	0	0	6 (20.7)	0
Group 3	3 (1.4)	0	1 (33.3)	0	**1 (33.3)**	1 (33.3)	0	0
Group 4	27 (12.2)	0	4 (14.9)	1 (3.7)	0	**11 (40.7)**	11 (40.7)	0
Group 5	55 (24.9)	0	5 (9.1)	3 (5.4)	0	2 (3.6)	**25 (45.5)**	20 (36.4)

According to 2018 ASCO/CAP guidelines, HER2 FISH status were divided into five groups in IHC 2+ patients: group 1, HER2/CEP17 ratio ≥ 2.0, average HER2 gene CN ≥ 4.0; group 2, HER2/CEP17 ratio ≥ 2.0, HER2 gene CN < 4.0; group 3, HER2/CEP17 ratio < 2.0, HER2 gene CN > 6.0; group 4, HER2/CEP17 ratio < 2.0, average HER2 gene CN ≥ 4.0 and ≤6.0; and group 5, HER2/CEP17 ratio < 2.0, HER2 gene CN < 4.0.

Bold values indicate statistical significance *p* < 0.05.

*IHC* immunohistochemistry.

Focusing on the average *HER2* gene and CEP17 CNs, we evaluated HER2 discordance between pre- and post-treatment specimens among the IHC 2+ tumours (Table 6). Of the 23 patients that changed HER2 category from IHC2+/*HER2* amplified to IHC2+/*HER2* non-amplified, 15 (65.2%) maintained the same *HER2* gene CN, whereas 8 (34.8%) showed a decreased *HER2* gene CN. In addition, chr 17 status was divided into m17, n17 and p17 according to CEP17 CN. Of the previously mentioned 23 patients, 17 (73.9%) maintained the same chr 17 status, while 2 (8.7%) patients with n17 before treatment changed to p17 after treatment. For comparison 13 patients that changed category from an IHC2+/*HER2* non-amplified to IHC2+/*HER2* amplified were assessed; of the 13 patients, 10 (76.9%) maintained the same *HER2* gene CN, whereas 3 (23.1%) showed an increased *HER2* gene CN. According to CEP17 CN, of the 13 patients, 10 (76.9%) maintained the same chr 17 status, while 1 (7.7%) with n17 before treatment changed to m17.

**Table 6:** Correlation between *HER2* gene CN and CEP17 CN in pre-treatment core biopsy and the post-treatment excision specimen among HER2 discordant patients.

Pre-treatment	Post-treatment					
	HER2 discordance (*HER2* amplified to non-amplified) (*n* = 23)			HER2 discordance (*HER2* non-amplified to amplified) (*n* = 13)		
	*HER2* gene CN			*HER2* gene CN		
*HER2* gene CN	<6.0 No. (%)		≥6.0 No. (%)	<6.0 No. (%)		≥6.0 No. (%)
<6.0	15 (65.2)		0	10 (76.9)		3 (23.1)
≥6.0	8 (34.8)		0	0		0

	CEP17 CN			CEP17 CN		
CEP17 CN	m17 (<1.5) No. (%)	n17 (≥1.5, <3.0) No. (%)	p17 (≥3.0) No. (%)	m17 (<1.5) No. (%)	n17 (≥1.5, <3.0) No. (%)	p17 (≥3.0) No. (%)
m17 (<1.5)	1 (4.3)	2 (8.7)	0	1 (7.7)	1 (7.7)	0
n17 (≥1.5, <3.0)	0	15 (65.2)	2 (8.7)	1 (7.7)	9 (69.2)	0
p17 (≥3.0)	0	2 (8.7)	1 (4.3)	0	1 (7.7)	0

*CN* copy number, *m17* monosomy chromosome 17, *n17* normal chromosome 17, *p17* polysomy chromosome 17.

## Discussion

In the current study, we aimed to identify predictive factors for pCR after NACT across different HER2-positive categories. We showed that HER2 IHC 3+ invasive BC had a higher pCR rate than IHC 2+*/HER2* amplified tumours when anti-HER2 therapy was received, consistent with earlier analyses [9]. In our study, pCR rate of HER2 IHC 3+ tumours following anti-HER2 therapy was 52%, within the range reported in clinical trials with similar treatment regimens; for example, pCR rate was 38% in the NOAH trial using NACT with trastuzumab, 39% in the NeoSphere and 58% in the TRYPHAENA trials using NACT with trastuzumab and pertuzumab [10, 26, 27]. In keeping with the significant impact on pCR that dual anti-HER2 therapy has shown [26–28], we also identified a higher pCR rate for NACT with dual anti-HER2 therapy compared to NACT with single ani-HER2 therapy in patients with IHC 3+ BC. Notably, the pCR rates among HER2 IHC 2+/*HER2* amplified tumours in patients who received anti-HER2 therapy were 20% in Group 1, 21% in Group 2 and 14% in Group 3, lower than the clinical trials. Regardless of ASCO/CAP FISH groups, IHC 2+/*HER2* amplified tumours showed significantly lower rates of pCR than IHC 3+ tumours. Consistent with previous studies, our data highlight that HER2 IHC 3+ and histological grade 3 are independent predictors of pCR following treatment with anti-HER2 therapy [9, 29]. Although a higher rate of pCR in HER2 IHC 3+ tumours was reported in the Krystel–Whittemore study [9], their cohort had higher proportions of histological grade 3, ER negative and patients who received dual anti-HER2 therapy. This is in keeping with our analysis of the HER2 IHC 3+ tumours that confirmed that a higher pCR rate was seen in histological grade 3 tumours treated with NACT and dual anti-HER2 therapy (64%).

Overall, in the HER2 IHC 2+/*HER2* amplified group treated with NACT and/or anti-HER2 therapy, ER-negativity was an independent predictor of pCR. Furthermore, we identified independent predictors of pCR by ASCO/CAP FISH group treated with NACT and/or anti-HER2 therapy: ER-negativity in Group 1 and histological grade 3 in Group 2. Predictors were not seen in the very small numbers of patients with tumours in Group 3. Indeed, because Groups 2, 3 and 4 are uncommon, the response of tumours in the different ASCO/CAP FISH groups to NACT has not been well explored [30]. Further analyses using a larger cohort are needed to validate these results, however our findings provide preliminary evidence that predictors of pCR differ between the ASCO/CAP FISH groups.

Hormone receptor (HR) status influences the response of a tumour to chemotherapy and the sensitivity of a tumour to combined chemotherapy and anti-HER2 therapy also differs according to HR status in the neoadjuvant setting [9–12, 31]. Because HER2-positive, HR-negative tumours are likely to be highly dependent on the *HER2* gene for growth, these tumours typically show a good response to anti-HER2 therapies [32]. In the present series, in patients who received anti-HER2 therapy, ER-negativity was significantly associated with pCR in univariate analysis, but was not an independent predictive factor in multivariate analysis. We observed that HER2 IHC 2+/*HER2* amplified ER-negative tumours showed a significantly higher rate of pCR than *HER2* amplified ER-positive tumours. Meanwhile, in HER2 IHC 3+ tumours neither ER-negative nor PR-negative subgroups had a significantly higher rate of pCR, similar to the findings of Miolo et al [33]. It has been previously reported that pathological characteristics differ between HER2 IHC 3+ and HER2 IHC 2+/*HER2* amplified tumours: IHC 3+ tend to be of higher histological grade, larger tumour size, and are often ER-negative and PR-negative [34]. These findings emphasise the importance of considering combined HER2 and HR status to select those patients most likely to benefit from neoadjuvant anti-HER2 therapy.

Despite lower rates of pCR in HER2-positive/HR-positive tumours, it has been reported that these tumours have a good prognosis, and that there is thus a weaker association between pCR and long-term outcome in HER2-positive/HR-positive tumours than HER2-positive/HR-negative tumours [8, 35]. However, neoadjuvant endocrine therapy for HER2-positive/HR-positive patients has not resulted in a marked improvement in pCR rate [35]. Further strategies are required to increase the pCR rate and improve outcome in patients with HER2-positive/HR-positive BC.

Our results again highlight variation in HER2, ER and PR status in some cancers before and after neoadjuvant treatment, although the reported frequency of this varies [13–18]. The changes in HER2 status with loss of HER2 expression in the post-treatment specimens was statistically significant. This may reflect the response of the HER2 positive clone in the tumour to NACT and/or anti-HER2 therapy, leaving the HER2 negative clone as a residual component. Similarly, the acquisition of HER2 positivity post treatment in a few cases is likely to reflect heterogeneity of HER2 expression. Of note, however, there was less frequent change in HER2 status following neoadjuvant NACT and/or anti-HER2 therapy in cases defined as definitely positive by IHC (HER2 IHC 3+) on pre-treatment specimens, compared to those that were IHC 2+. It is unclear whether loss of *HER2* amplification reflects response to therapy, a mechanism of resistance or heterogeneity of HER2 expression [14]. If such changes in HER2 status will affect post-neoadjuvant treatment decisions, such as the tailoring of subsequent adjuvant therapy, based on these results, we suggest that HER2 should be re-tested in post-neoadjuvant surgical specimens, particularly in HER2 2+/*HER2* amplified tumours. Moreover, it has been reported that such alterations provide prognostic information with loss of HER2-positivity in residual tumours after neoadjuvant treatment shown to be associated with a poorer outcome compared with tumours with preserved HER2-positive status [16–18].

Our study has some limitations. First, this was a retrospective non-randomised study, and our samples were collected from multiple institutions which may have some selection bias effect. Second, some subset analyses were underpowered to detect subgroup differences due to small sample size, especially those in HER2 2+/FISH Group 3 BC (*n* = 9). Third, because of differences in regimens of chemotherapy prescribed, the influence of those on the different HER2-positive groups of BC with respect to pCR needs further study. Finally, not all patients with HER2-positive tumours received anti-HER2 therapy in this cohort; some patients, including some older women with co-morbidities and those with small and node-negative HER2-positive tumours, were less likely to receive neoadjuvant anti-HER2 therapy. In our cohort, there were no significantly differences in clinicopathological features such as age, HR status and histological grade whether anti-HER2 therapy given or not (Table 7). Special tumour type was a high percentage in patients received chemotherapy alone (*p* = 0.04).

**Table 7:** HER2-positive patient baseline characteristics between neoadjuvant chemotherapy alone and anti-HER2 therapy given.

Characteristic	Neoadjuvant treatments		*P* value
	Chemotherapy alone (*n* = 32) No. (%)	anti-HER2 therapy given (*n* = 317) No. (%)	
Age			
Median [range]	53 [29–80]	52 [23–86]	
Histology type			
Ductal, NST	24 (75.0)	285 (89.9)	**0.04**
Special types	4 (12.5)	13 (4.1)	
Lobular	3 (9.4)	11 (3.5)	
Mixed (ductal and lobular)	0	5 (1.6)	
Unknown	1 (3.1)	3 (0.9)	
Histological grade			
1	1 (3.1)	3 (0.9)	0.18
2	21 (65.7)	166 (52.4)	
3	9 (28.1)	133 (42.0)	
Unknown	1 (3.1)	15 (4.7)	
ER			
Positive	23 (71.9)	211 (66.6)	0.69
Negative	9 (28.1)	104 (32.8)	
Unknown	0	2 (0.6)	
PR			
Positive	14 (43.7)	134 (42.3)	1
Negative	15 (46.9)	149 (47.0)	
Unknown	3 (9.4)	34 (10.7)	

Bold values indicate statistical significance *p* < 0.05.

*NST* no special type.

In conclusion, the data presented here indicate that the maximum benefit of neoadjuvant anti-HER2 therapy is observed in the subgroup of patients with tumours that are HER2 IHC 3+, histological grade 3 or IHC 2+/*HER2* amplification co-existing with ER-negativity. Among tumours that were HER2 IHC 2+/*HER2* amplified, the predictors of pCR were different in the various ASCO/CAP FISH groups. In our study, 22% of HER2-positive tumours before treatment changed to HER2-negative after neoadjuvant treatment, more commonly in HER2 IHC 2+/*HER2* amplified tumours, especially ASCO/CAP FISH Group 1. Reassessment of HER2 status following neoadjuvant treatment should be considered in patients in whom it will facilitate further management decisions.
